# The role of long noncoding RNAs in livestock adipose tissue deposition — A review

**DOI:** 10.5713/ab.21.0006

**Published:** 2021-04-23

**Authors:** Lixue Wang, Yuhuai Xie, Wei Chen, Yu Zhang, Yongqing Zeng

**Affiliations:** 1 Shandong Provincial Key Laboratory of Animal Biotechnology and Disease Control and Prevention, College of Animal Science and Technology, Shandong Agricultural University, Tai’an, Shandong 271018, China; 2 Department of Immunology, School of Basic Medical Sciences, Fudan University, Shanghai, 200032, China

**Keywords:** LncRNAs, Adipose Tissue Deposition, Livestock

## Abstract

With the development of sequencing technology, numerous, long noncoding RNAs (lncRNAs) have been discovered and annotated. Increasing evidence has shown that lncRNAs play an essential role in regulating many biological and pathological processes, especially in cancer. However, there have been few studies on the roles of lncRNAs in livestock production. In animal products, meat quality and lean percentage are vital economic traits closely related to adipose tissue deposition. However, adipose tissue accumulation is also a pivotal contributor to obesity, diabetes, atherosclerosis, and many other diseases, as demonstrated by human studies. In livestock production, the mechanism by which lncRNAs regulate adipose tissue deposition is still unclear. In addition, the phenomenon that different animal species have different adipose tissue accumulation abilities is not well understood. In this review, we summarize the characteristics of lncRNAs and their four functional archetypes and review the current knowledge about lncRNA functions in adipose tissue deposition in livestock species. This review could provide theoretical significance to explore the functional mechanisms of lncRNAs in adipose tissue accumulation in animals.

## INTRODUCTION

Adipose tissue deposition is a crucial economic index for livestock production evaluation and is closely associated with meat quality by influencing the lean meat ratio and intramuscular fat content [1,2]. However, in human medical studies, excess fat is a health concern, as abnormal adipose tissue accumulation might lead to obesity, diabetes, atherosclerosis, and other diseases [3,4]. In the early embryonic development period and the birth period, the number of adipocytes rapidly increases because of the impact of maternal genetic and nutritional factors. The volume of adipocytes increases relatively quickly in the late embryonic development and growth stage [5,6]. Subcutaneous, abdominal, bone, intermuscular and intramuscular tissues are the primary locations of animal adipose depots [7–9]. A high content of lipids in these sites can affect the lean meat ratio and meat quality of animals [10]. Meanwhile, it also leads to obesity and type 2 diabetes in humans [11–13].

Over the past few decades, with the rapid development and great benefits of next-generation sequencing (NGS) technologies, large-scale genome sequencing data can be rapidly generated, which has revolutionized our understanding of genetic informatics. In this field, long noncoding RNAs (lncRNAs) have emerged as important regulators of gene expression in diverse biological contexts. Increasing evidence has demonstrated that lncRNAs play important roles in animal breeding, reproduction and livestock production, especially in the breeding of high-quality meat-producing animals [14,15]. Evidence showed that lncRNAs could regulate muscle growth [16], adipogenic differentiation [17], and adipogenesis of porcine ontramuscular preadipocyte [18].

However, compared with their application in human biomedical studies, the application of lncRNAs in livestock production is much less common. Although previous reviews about lncRNAs in livestock have provided information about the identification, features, and genomic annotation of lncRNAs [19,20], some newer aspects of lncRNA function in improving livestock production have not yet been studied extensively. Thus, in this paper, we review the current studies about the roles of lncRNAs in adipose tissue deposition in livestock species, as well as lncRNA biology, which is expected to benefit animal and food biologists.

## CHARACTERISTICS OF LncRNAs

LncRNAs are defined as noncoding RNAs longer than 200 nucleotides, a cutoff that is used mainly to distinguish lncRNAs from small RNAs such as microRNAs (miRNAs) and transfer RNAs [21]. LncRNAs can be further classified into six different types, namely, long-intergenic ncRNAs (lincRNAs), intronic lncRNAs, antisense lncRNAs, sense lncRNAs, enhancer-associated lncRNAs, and circular lncRNAs [22]. Similar to messenger RNA (mRNAs), lncRNAs are transcribed by RNA polymerase II (Pol II) from genomic loci with chromatin states similar to those of mRNAs. In most instances, lncRNAs are biochemically indistinguishable from mRNAs except for their lack of a translated open reading frame [23]. However, differences have been observed in some lncRNAs that contain introns, can be nonpolyadenylated or are bidirectionally transcribed [24]. Homology and conservation are also important indicators in studying the biological function and relationship with protein-coding genes of lncRNAs.

Typically, evolutionary conservation refers to sequence conservation, which is determined based on similarities between nucleotide or amino acid sequences. A comparison of mammalian transcripts and lncRNAs found in zebrafish revealed that the few instances of significantly conserved sequences were mostly limited to short sequence stretches. Hence, lncRNAs evolve rapidly and often lack orthologs in other species [25]. Secondary and tertiary structures are vital for RNA function [26]. Studies have demonstrated the existence of structured regions within lncRNAs, and multiple sequence alignments have supported the presence of conserved structures [27,28]. In addition, while the process of lncRNA production from specific genomic sites may be conserved, the sequence, structure, and function of the products may not be conserved [29]. Finally, the conservation of lncRNA functions in different species is also a relevant point in the field, and is often associated with sequence and structure [30]. Most importantly, the low sequence conservation of lncRNAs across different species has led some to dismiss lncRNAs as transcriptional noise [31]. Despite many predictions from RNA sequencing (RNA-seq) data, few lncRNA orthologs that function across species have been experimentally verified. In addition, data from the UCSC Genome Browser (http://genome.ucsc.edu/) and NONCODE database (http://www.noncode.org/) can help us identify conserved lncRNAs across different species.

Based on transcriptome-wide methods, lncRNAs generally exhibit more specific expression profiles than mRNAs [32]. That is, lncRNA expression is more specific to different cells, tissues and stages of development, and this specific expression can be more tightly regulated than the expression of protein-coding genes. Furthermore, lncRNA expression is correlated with mRNA expression both in *cis* and in *trans*, suggesting that lncRNAs may be coregulated in expression networks [23,33]. Many research findings have indicated an essential role of lncRNAs in regulating the development and maintenance of endocrine organs and hormonal signaling, and misregulation of these processes can lead to disease [34]. Because of poor conservation and specific expression, the mechanisms of lncRNAs in regulating biological processes are complex and unique. LncRNAs can direct elements in *cis* or in *trans* elements to bind to genes or ribosomal protein complexes, thereby regulating the expression of adjacent or distant genes and acting as molecular guides [35]. In addition, lncRNAs can act as a central platform in different related types of macromolecular components and can play a regulatory role in a variety of biological signal transduction processes.

The function of lncRNAs is associated with their unique subcellular localization patterns. The localization of lncRNAs, mainly nuclear and cytoplasmic localization, has recently become a hot topic in the field. Evidence has shown that compared with mRNAs, lncRNAs are more enriched in the nucleus relative to the cytoplasm [31]. RNA fluorescence *in situ* hybridization (RNA FISH) has been used to evaluate lncRNA showed the lncRNAs localization, which showed that lncRNAs exhibit many different subcellular localization patterns ranging from defined subnuclear points and nuclear retention to diffuse whole-cell spread. They do not categorically occupy one particular location; rather, lncRNAs are ubiquitous [36].

## MECHANISMS OF ADIPOSE TISSUE DEPOSITION

Adipocytes are derived from multipotent stem cells that are fibroblast-like mesenchymal precursors and can differentiate into adipocytes, myoblasts, osteoblasts, and chondrocytes after receiving signals from specific transcription factors [37, 38]. After signaling by bone morphogenetic protein 4 (BMP4), multipotent fibroblast-like stem cells commit to the adipocyte lineage, and finally differentiate into mature adipocytes [39]. The proliferation and hypertrophy of adipocytes are the main factors causing adipose tissue deposition. In pigs and ruminants, hypertrophic adipose tissue predominates after birth. The postnatal growth of adipose tissue results from an increase in the number (hyperplasia) and especially the size (hypertrophy) of adipocytes [40]. Previously, a detailed and systematic endeavor was undertaken to define the transcriptional events regulating preadipocyte differentiation (adipogenesis) and adipocyte function. Peroxisome proliferator-activated receptor γ (PPARγ) and CCAAT enhancer binding proteins (C/EBPα) are the master regulators of adipogenesis, which has been confirmed by the vast majority of *in vivo* and *in vitro* studies [41,42]. Moreover, other transcription factors ultimately affect adipose tissue deposition through PPARγ and C/EBPα [43,44].

Many signaling pathways modulate the process of preadipocyte differentiation into mature adipocytes. BMPs are a class of conserved signaling molecules in the transforming growth factor-β family of proteins. BMP signaling through BMP receptors results in the intracellular phosphorylation and activation of SMAD proteins, which causes the transcription of PPARγ and promotes adipogenesis [45]. The Wnt signaling pathway was once thought to be a negative regulator of adipose differentiation. However, wnt5b could partially inhibit the canonical Wnt/β-catenin signaling pathway and promote adipocyte differentiation [46]. In addition, the hedgehog [47], and notch [48] signaling pathways could also regulate adipose tissue deposition.

## THE FUNCTION OF LncRNAs IN ADIPOSE TISSUE DEPOSITION

LncRNAs are involved in the mechanisms of adipose tissue deposition by influencing the adipogenesis and lipid metabolism. On the one hand, the proliferation and hypertrophy of adipocytes are the main processes of adipogenesis. Abundant evidence indicates that lncRNAs play an important role in these biological processes of adipose tissue accumulation. Many lncRNAs have been shown to influence adipocyte differentiation; for example, lncRNA_000414 inhibits the proliferation of intramuscular adipocytes [49]. Lnc-U90926 was found to be predominantly expressed in adipose tissue and was mainly located in the cytoplasm. The expression of Lnc-U90926 inhibited 3T3-L1 preadipocyte differentiation to adipose tissue by decreasing the mRNA levels of PPARγ2, adipose tissue acid binding protein 4 (FABP4), and adiponectin (AdipoQ), and the protein levels of PPARγ and FABP4 [50].

On the other hand, lipid metabolism can be considered an essential factor affecting adipose tissue deposition. Numerous lncRNAs have been recognized as a novel regulators of fat metabolism. A recent study showed that the lncRNA *SRA* limits adipose triglyceride lipase promoter activity in hepatic steatosis primarily by inhibiting forkhead box protein O1 expression [51]. The overexpression of *lncHR1* blocked the expression of sterol regulatory element binding protein (SREBP-1c) and fatty acid synthase and suppressed the accumulation of triglycerides and lipid droplets in hepatocytes induced by oleic acid [52]. Evidence also showed that *lncSHGL* could recruit heterogeneous nuclear ribonucleoprotein A1 to increase the level of calmodulin protein without affecting its transcription. Thus, *lncSHGL* activated the phosphatidylinositol 3-kinase (PI3K)/Akt pathway and inhibited the mammalian target of rapamycin (mTOR)/SREBP-1C pathway to regulate hepatic glucose/lipid metabolism [53].

Although only a few functional lncRNAs have been well characterized so far, they have been shown to regulate the majority of gene expression programs in adipose tissue deposition [54]. Moreover, lncRNAs also regulate chromatin-modifying proteins, transcription, and translation processes. There are four archetypes of lncRNA functions, as detailed below. In addition, studies also have shown that lncRNAs may fulfill several archetypes to regulate adipose tissue accumulation (Table 1).

### Signal

**Figure 1:**
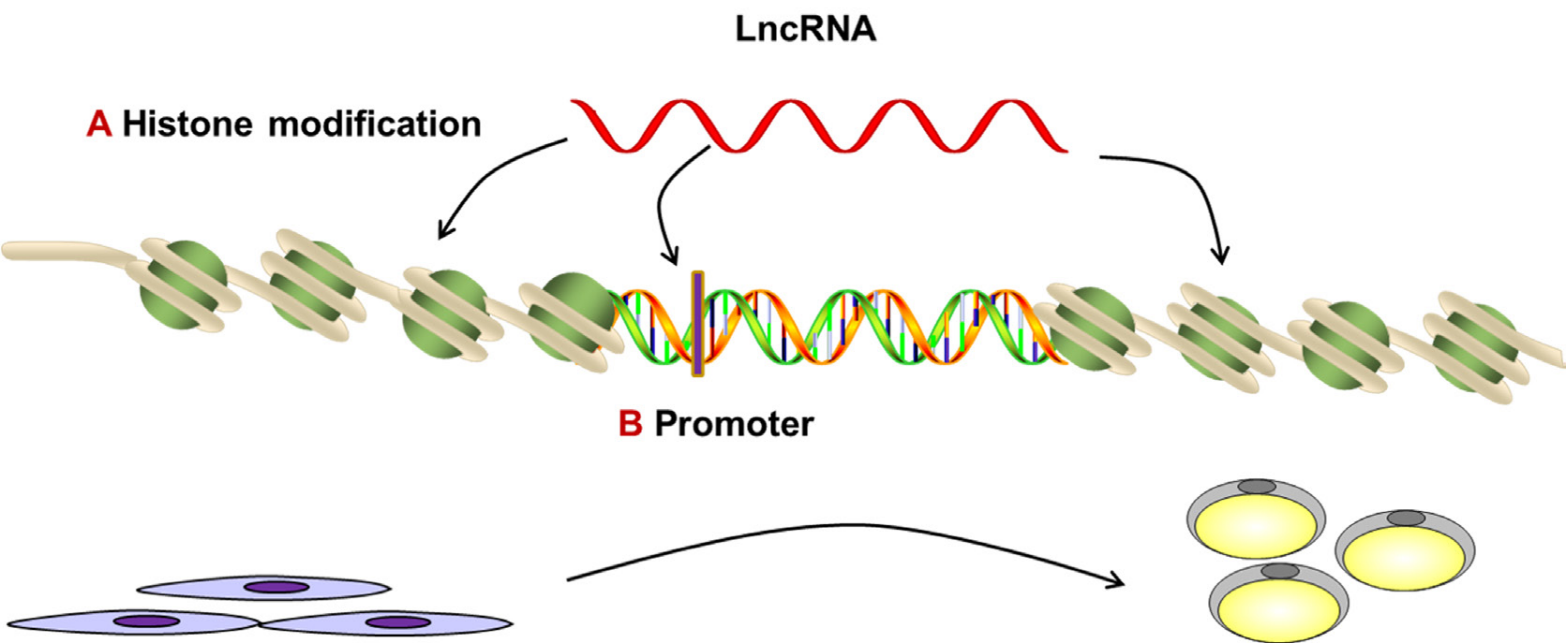
Long noncoding RNAs regulate the expression of adipogenic genes by modifying of chromatin.

The first mode of action of lncRNAs is to regulate the transcription of downstream genes as molecular signals. LncRNAs can act as molecular signals because their expression is more cell-, tissue- and developmental stage-specific. The transcription of individual lncRNAs occurs at a very specific time and place to integrate developmental cues, interpret cellular context, or respond to diverse stimuli. Many studies have shown that lncRNAs can act as signals to regulate adipocytes differentiation and lipid metabolism. The lncRNA miR-31 host gene contributes to histone H3 lysine 4 trimethylation (H3K4me3) and H3 acetylation to promote the transcription of FABP4, which is an important molecule in the induction of adipocyte differentiation [55]. Similarly, it has been reported that the lncRNA adipogenic differentiation-induced noncoding RNA (*ADINR*) can increase H3K4me3 and decrease histone 3 lysine 27 trimethylation (H3K27me3) histone modifications in the C/EBPα locus during adipogenesis by specifically binding to PA1 and recruiting MLL3/4 histone methyltransferase complexes. This indicated that *ADINR* might play vital roles in regulating the differentiation of human mesenchymal stem cells (MSCs) into adipocytes by modulating C/EBPα in *cis* [56]. These findings suggest that lncRNAs are involved in the expression of adipose tissue-related genes through the regulation of histones (Figure 1A). In addition, lncRNAs can regulate the transcription factors associated with adipose tissue deposition by binding to their promoters of these transcription factors. For example, bovine adipocyte differentiation-related long noncoding RNA 1 significantly inhibited the enhanced expression of mitochondrial protein glutaredoxin 5 promoter activity through C/EBPα [57] (Figure 1B). These studies demonstrated that lncRNAs can act as molecular signals by directly regulating histone modification or the promoters of transcription factors. In addition, these lncRNAs can serve as biomarkers of functionally significant biological events because the chromatin state and the expression of regulatory elements can be easily inferred by the expression of their related lncRNAs. Chromatin immunoprecipitation (ChIP) and chromatin isolation by RNA purification (CHIRP) assays are generally required for the functional verification of these lncRNAs in the modulation of histones.

### Decoy

**Figure 2:**
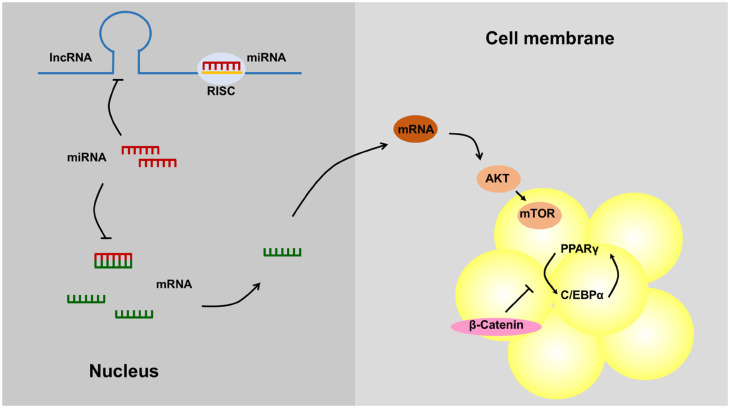
LncRNAs act as ceRNA to regulate adipogenesis. LncRNAs competitively bind to miRNAs, which increases levels of the mRNAs originally bound to miRNAs and thus regulates adipose tissue deposition (lncRNA expression in the nucleus is indicated as an example). LncRNAs, long noncoding RNAs; ceRNA, competing endogenous RNAs; miRNAs, microRNAs; mRNAs, messenger RNA.

The second action mode of lncRNAs is interference. LncRNAs can directly bind to transcription factors to then regulate downstream gene transcription. This mainly represents a molecular decoy mechanism, in which the lncRNA acts as a “molecular sink” for the transcription factors. LncRNAs acting as decoys of miRNAs have been called competing endogenous RNAs (ceRNAs), and the ceRNA mechanism is a theoretical hypothesis of RNA expression regulation. Endogenous lncRNAs can act as ceRNAs by competing with the seed sequences of miRNAs to release related mRNAs from miRNA-mediated inhibition. This is also the most common pattern of lncRNA interaction with each other [63] (Figure 2). For example, LRP6 is the direct target of *miR-21* and is required for *miR-21*-induced intracellular lipid accumulation. Research has shown that the lncRNA maternally expressed gene 3 (lncRNA *MEG3*) is considered a ceRNA that regulates hepatic lipogenesis by competitively binding to *miR-21* with LRP6 through the mTOR signaling pathway to alleviate lipid over-deposition [15]. The LncRNA growth arrest-specific transcript 5 can regulate the adipogenic differentiation of MSCs by acting as a ceRNA to sponge miR-18a [14]. Similarly, adipocyte differentiation-associated long noncoding RNA could act as a ceRNA to interact with miR-204 and inhibit adipocyte differentiation by regulating sirtuin 1 [58]. The LncRNA terminal differentiation-induced ncRNA also served as a sponge of miR-31, which can directly target C/EBPα to modulate adipogenic differentiation in human adipose tissue-derived from MSCs [59].

### Guide

**Figure 3:**
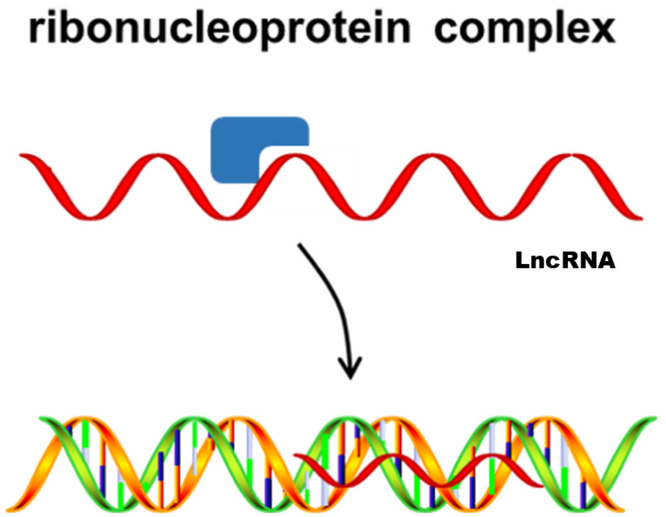
Long noncoding RNAs act as a guide to direct the localization of ribonucleoprotein complexes to specific targets.

Third, lncRNAs act as guides to bind proteins and then direct the localization of the ribonucleoprotein complex to specific targets. This transcriptional regulation mediated by lncRNAs can be *cis*-acting or *trans*-acting (Figure 3). Evidence has shown that *ADINR* can specifically bind to PA1, recruiting MLL3/4 histone methyltransferase complexes and then activating C/EBPα transcription in cis to promote adipogenesis [56]. Heterogeneous nuclear ribonucleoprotein U (hnRNPU) is required for brown adipocyte development, and brown adipose tissue-specific lncRNA 1 (lnc *BATE1*) can interact with hnRNPU to modulate brown adipogenesis by acting in *trans* [60].

### Scaffold

**Figure 4:**
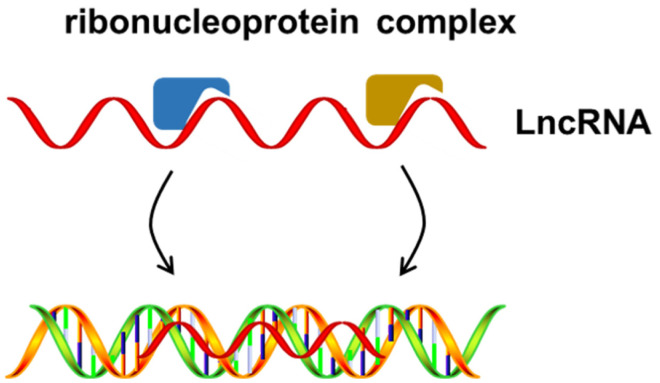
Long noncoding RNAs act as scaffolds that bring together multiple proteins to form ribonucleoprotein complexes.

Finally, lncRNAs have different binding domains for different effector molecules, which makes it possible to bind multiple effectors simultaneously. Thus, lncRNAs could induce transcriptional activation or inhibition in time and space by interacting with different effectors. Scaffolds might be the most functionally intricate and complex regulatory mechanism of lncRNAs, in which lncRNAs act as central platforms to affect the regulation of multiple signaling pathways (Figure 4). *H19* (a lncRNA transcribed from the H19 gene) is a classic lncRNA that regulates ligand-dependent corepressor to regulate the balance between osteogenic and adipogenic differentiation of bone marrow stromal stem cells in mice by sponging miR-188 [61]. In addition, *H19* can bind with methyltransferase MBD1 to form H19-MBD1 complexes in mature brown adipocytes, potentially via MBD1-dependent alterations of H3K9me3 KMT recruitment. In these two studies, *H19* was proposed as a paternally expressed gene (PEG) gatekeeper in brown adipocytes and was shown to repress brown adipose tissues PEG by recruiting MBD1 chromatin modifiers [62].

## STUDIES OF LncRNAs IN ADIPOSE TISSUE DEPOSITION OF LIVESTOCK

Compared with clinical studies in humans, studies of the function of lncRNAs in adipose tissue deposition and meat quality in livestock have been limited. Many transcriptome sequencing studies on adipose tissue accumulation in different species of livestock have been published. However, most of these studies focused on screening differentially expressed genes (DEGs) using RNA-seq without sufficient exploration of the function and mechanisms of the DEGs. In previous papers, a number of lncRNAs have been well characterized to be involved in adipose tissue accumulation in cells or rodents [50,64]. These studies could provide insights into the important role and application of lncRNAs in regulating the development of adipose tissue in farm animals. Although the conservation of lncRNAs is lower than that of other ncRNAs and protein-coding RNAs, the functions of lncRNAs are important [65]. Thus, studying the conservation of lncRNAs among different species seems to be significant for farm animal genomic and transcriptomic studies, such as those focusing on adipose tissue deposition and fat metabolism. When a certain lncRNA is well conserved in different species such as humans, mice, pigs, and cattle, the known mechanism of action and function of this lncRNA might be applicable to other animals. Therefore, research on lncRNA conservation among species might be valuable for the research or application of conserved lncRNAs in different animals, especially domestic large livestock animals.

### Cattle

Intramuscular fat deposition in cattle is of economic importance and has been explored in several researches. In livestock, adipose tissue and muscle development are closely related to body weight, marbling content, and meat quality, especially marble beef yield [66]. High-throughput sequencing was performed on six calf and adult bovine adipose tissues from Chinese Qinchuan cattle to select candidate lncRNAs associated with adipose tissue accumulation and muscle development [67]. The researchers also focused on identifying DEGs between muscle and adipose tissues to determine their effects on intramuscular fat and meat quality. Hanwoo cattle (*Bos taurus coreanae*) are famous for thier high marbling content and relatively thin muscle fibers in Korea. A previous study profiled tissue-specific lncRNAs by using comparative analysis of muscle and other adipose tissues (intramuscular, subcutaneous, and omental) in Hanwoo cattle, and 76 lncRNAs were identified to be associated with marbling content and meat quality [68]. In livestock production, a novel lncRNA mainly localized to the nucleus and named miR-221 host gene (*MIR221HG*) because it overlaps with miR-221 in the genome was proven to regulate bovine adipocyte differentiation [69]. Studies found that the expression of lnc *FAM200B* in bovines had a significant positive correlation with *C/EBPα* expression, suggesting that lnc *FAM200B* might participate in the regulation of adipocyte development [70]. In addition, evidence indicated that *PSXV-9*, a novel antisense lncRNA of *C/EBPα*, could inhibit bovine adipogenic differentiation [71].

### Pigs

Domestic pigs (*Sus scrofa domesticus*) are one of the major animal models of obesity and adipose tissue deposition [63,72]. Similar to studies on lncRNAs in cattle, there are also numerous high-throughput sequencing studies comparing different pig species. For example, researchers found DEGs between Jinhua and Landrace pigs [73] and Laiwu and Large White pigs [74] and screened candidate lncRNAs associated with adipose tissue accumulation. Evidence has shown that the lncRNA *IMF4* regulates porcine intramuscular preadipocyte adipogenesis by attenuating autophagy to inhibit lipolysis [18]. *PU.1*, also known as spleen focus forming virus proviral integration oncogene spi1, inhibits adipogenesis. In addition, the *PU.1* antisense lncRNA (*PU.1 AS* lncRNA) promotes adipogenesis through the formation of a sense-anti-sense RNA duplex with *PU.1* mRNA [75]. Similarly, *PLA2G16-AS* and PLA2G16 were also reported to be expressed in pork adipose tissues [76]. Besides, the lncRNA *IMFlnc1*, a differentially expressed lncRNA identified through sequencing between Huainan and large white pigs, promoted porcine intramuscular adipocyte adipogenesis by sponging miR-199a-5p and upregulating caveolin-1 expression [77].

### Other livestock

There have also been abundant studies about adipose tissue deposition based on high-throughput sequencing data from chickens [78], rabbits [79], sheep [80] and other kinds of livestock. Evidence also showed that the lncRNA intramuscular fat-associated long non-coding RNA promoted intramuscular adipocyte differentiation by sponging miR-128-3p and miR-27b-3p in chicken which targeted PPARγ [81]. Although these studies have identified differentially expressed candidate lncRNAs related to adipose tissue accumulation, there are still some drawbacks. First, these studies used experimental samples that might not have been considered the genetic background. In addition, the screened differentially expressed lncRNAs were not well explored, and the mechanism of regulation of adipose tissue deposition by these potential lncRNAs is unclear. Therefore, research methods to explore lncRNA functions in adipose tissue accumulation have important future implications.

## PERSPECTIVES AND FUTURE OPPORTUNITIES

To date, differential adipose tissue deposition abilities have been observed in different breeds and even the different individuals under the same genetic background, and the mechanism of this phenomenon is still unknown. The key developmental period and physiological and biochemical mechanisms of adipose tissue accumulation still need to be further explored. In addition, research on the interaction and regulation between muscle and intramuscular adipose tissue is an important aspect to improve the meat quality of livestock production. It is essential to explore this regulatory mechanism for its implementation and application in meat production in order to improve livestock productivity and the development of high-quality ecological products.

Compared to methods involving biologics or small molecules, RNA-based breeding and production are characterized by unique and important benefits and challenges that should be taken into consideration. RNA methods are based on nucleotide hybridization to interact with the targets and include identification of the sequence and testing of candidate genes for activity. Despite these advantages, mechanisms of lncRNA regulation of adipose tissue deposition have to be characterized and optimized, which can often be a challenging process. Although lncRNA applications in farm animals are still at an early stage, it is expected that production based on the involvement of lncRNAs in adipose tissue deposition and pathway regulation will one day be achieved. Expanded lncRNA research in livestock species will provide new fundamental insights into the complexity of gene regulation of important biological processes. For the identification of candidate biomarkers, focusing on lineage-specific lncRNAs seems to be the ideal approach, as is the case with the lncRNA *MEG3* mentioned above [15]. Therefore, researchers should pay attention to the conservation of lncRNAs before starting experiments. In addition, the level of lncRNA expression is also a key factor for developing lncRNA based predictions. Overall, lncRNAs are a promising tool that can enable researchers to discover molecular signatures that help to improve meat quality for fam animals in the future. Given the conservation and specific expression of lncRNAs and its response to adipose tissue deposition, the lncRNA class deserves further investigation into their physiological roles and molecular mechanisms of action, which finally, will contribute to better explain the whole phenotypic variation of adipose tissue accumulation and basic biological processes in livestock animals.

## Figures and Tables

**Table 1:** LncRNAs function in adipose tissue deposition

Function archetype	LncRNA acronym	Full name	Function	References
Signal	MIR31HG	miRNA-31 host gene	Contribute to H3K4me3 and H3 acetylation	[55]
	ADINR	Adipogenic differentiation induced noncoding RNA	Function in histone modification during adipogenesis	[56]
	BADLNCR1	Bovine adipocyte differentiation related long non-coding RNA 1	Inhibit mitochondrial protein GLRX5 promoter activity	[57]
Decoy	MEG3	Maternally expressed 3	Activate the mTOR signaling pathway to alleviate lipid overdeposition	[15]
	GAS5	Growth arrest specific 5	Regulate adipocyte differentiation	[14]
	ADNCR	Adipocyte differentiation-associated long noncoding RNA	Inhibit adipocyte differentiation by regulating SIRT1	[58]
	TINCR	Terminal differentiation-induced ncRNA	Modulate adipocyte differentiation of adipose tissue-derived MSCs	[59]
Guide	ADINR	Adipogenic differentiation induced noncoding RNA	Activate C/EBPα transcription in cis to promote adipogenesis	[56]
	BATE1	Brown adipose tissue enriched long noncoding RNA 1	Interact with hnRNPU to modulate brown adipogenesis by acting in trans	[60]
Scaffold	H19	lncRNA transcribed by the H19 gene	Regulate the balance between osteogenic and adipogenic differentiation	[61,62]
